# UV light-blocking contact lenses protect against short-term UVB-induced limbal stem cell niche damage and inflammation

**DOI:** 10.1038/s41598-018-30021-8

**Published:** 2018-08-22

**Authors:** M. Notara, S. Behboudifard, M. A. Kluth, C. Maßlo, C. Ganss, M. H. Frank, B. Schumacher, C. Cursiefen

**Affiliations:** 1 Department of Ophthalmology, University of Cologne, Cologne, Germany; 2 TICEBA GmbH, Im Neuenheimer Feld 517, Heidelberg, Germany; 3 RHEACELL GmbH & Co. KG, Im Neuenheimer Feld 517, Heidelberg, Germany; 4 Transplant Research Program, Boston Children’s Hospital, Harvard Medical School, Boston, MA USA; 5 Harvard Stem Cell Institute, Harvard University, Cambridge, MA USA; 6 School of Medical Sciences, Edith Cowan University, Joondalup, WA Australia; 7 Institute for Genome Stability in Ageing and Disease, CECAD Research Center, Joseph-Stelzmann-Str. 26, 50931 Cologne, Germany; 8 Center for Molecular Medicine Cologne (CMMK), University of Cologne, Cologne, Germany

## Abstract

UVB irradiation has been linked to pathogenesis of pterygium, a conjunctival tumor growing onto transparent cornea, the windscreen of the eye. Due to corneal anatomy, ambient UVB irradiation is amplified at the stem cell-containing nasal limbus. The aim of this study was to analyse the effect of a UV-blocking contact lens (UVBCL, senofilcon A, Class 1 UV blocker) on limbal epithelial cells and fibroblasts under UVB irradiation compared to a non-UVB-blocking contact lens. UVBCL prevented UVB-induced DNA damage (as assessed by cyclobutane pyrimidine dimer immunostaining) as well as a decrease in proliferation and scratch wound closure rate of both limbal epithelial and fibroblast cells. Similarly, UVBCL protected limbal epithelial cells from UVB-induced loss of their phenotype in terms of colony forming efficiency and stem cell marker expression (ABCB5, P63α, integrin β1) compared to controls. Moreover, with UVBCL pro-inflammatory cytokines such as TNFα and MCP1 remained unchanged. These data demonstrate the significance of UV-protection in preserving the limbal niche in response to at least short-term UVB. Our data support the use of UVBCL in protecting limbal niche cells, especially after limbal stem cell transplantation and in patients after pterygium surgery, to help prevent recurrences.

## Introduction

The use of protective eyewear such as sunglasses and, if needed, UV blocking contact lenses against UV radiation has previously been recommended as a prophylactic measure against UV-induced eye damage^1^. UV-blocking contact lenses (UVBCL) have been proven preventative against acute photo-keratitis caused by UV overdoses in rabbit models^2,3^. However, their specific benefit in maintaining the phenotype and functionality of corneal cell populations and especially limbal epithelial stem cells has yet to be investigated.

The cornea is susceptible to UV irradiation due to its exposed position at the front of the eye, its shape and its natural transparency, which lead to a peripheral UV-focusing effect on the nasal limbus. There, the UV irradiation is amplified by a factor of 20^4,5^. This is the typical site for the onset of pterygium, a benign but sight-threatening vascularised tumour whose pathogenesis is strongly linked to UV exposure and which is expanding on the corneal equator leading to discomfort and decrease or loss of vision^6^.

As such dramatic phenotypic changes occur in the limbus and its adjacent tissues, changes in the limbal stem cell niche which contain a population of limbal epithelial stem cells (LESC) are also expected. LESCs play a fundamental role in the maintenance of corneal clarity by maintaininging its epithelium^7^. Histological evidence demonstrate that responsible for pterygium onset is a limbal epithelial cell able to express matrix metalloproteinases (MMPs)^8,9^, and basal limbal markers suggest that the condition may indeed be a limbal stem cell disorder^10^. However, the precise of LESC in pterygium pathogenesis as well as the specific effect of chronic UV irradiation on these stem cells remain largely unknown. In addition, UV damage on LESC niche accessory cells including limbal fibroblasts (HLF) may compromise the good function of the niche. In this regard, long term protection of the limbal niche and its resident LESCs from chronic UV irradiation could lead to disease prevention and contribute to their better function as key contributors to corneal homeostasis.

Chronic UV exposure can induce extensive alterations linked to pterygium etiology. Signs of DNA damage have been detected in pterygium either through formation of base dimers following direct absorption of the UV light by DNA or indirectly via by-products of UV-induced oxidative stress^11^. Also, UV-induced cornea alterations are regulated by the increased expression of pro-inflammatory interleukins^12,13^ and tumour necrosis factor alpha (TNFα)^14^, which associate with the inflammatory cell migration linked to pterygium. Furthermore, growth factors such as vascular endothelial growth factor (VEGF)^15,16^, and VEGF-C^15^ are also increased. This upregulation relates to the higher density of lymphatic vessels and vascular networks linked to pterygium recurrence and staging^17,18^. Collectively, changes in the above factors mediate UV-induced inflammation, neovascularisation, hyperplasia and tissue remodelling associated with pterygium and have been observed post UV radiation in normal cornea, conjunctiva and pterygium specimens as well as in isolated and cultured cells^13,19^. Thus far, the effectiveness of UVBCL against these changes has not been reported.

Assessment of the protective effect of UV-blocking contact lens wear on corneo-limbal cellular phenotype, DNA damage or cytokine expression is not practical in a clinical setting. An *in vitro* assessment of human primary cells and tissue samples has yet to be reported. The present study directly investigated for the first time the effect of UVB on LESCs and the LESC niche phenotype while assessing the protective role of UV-blocking contact lenses on the stem cell population and on changes in the corneo-limbal environment that are linked to increased corneal vascularization and pterygium development and recurrence.

## Materials and Methods

### Culturing of 3T3 mouse fibroblasts

A 3T3 mouse fibroblast cell line, a gift from the lab of Professor Nischt (Department of Dermatology, Uniklinik Köln, Cologne, Germany) was cultured in Dulbecco’s Modified Eagle Medium (DMEM, life technologies, Darmstadt, Germany) with added with 10% Fetal Bovine Serum (Gibco, Darmstadt, Germany) and 1% penicillin/streptomycin/amphotericin (Life Technologies, Darmstadt, Germany). Culture medium was exchanged every 48 h and the cells were passaged at a ratio of 1:10 upon reaching 60-70% confluence. The cultures were maintained at 37 °C and 5% CO_2_ in air. Prior to use as a feeder layer for limbal epithelial cells, the fibroblasts were treated with culture medium containing 6 μg/ml mitomycin C (Sigma, Munich, Germany) for 3 hours in order to cause their growth arrest.

### Primary human limbal epithelial cell harvesting and maintenance

Limbal epithelial cells were isolated from human corneo-scleral rims and corneal buttons, a surplus of surgery. Ethics Approval (State of Cologne Ethics Approval Committee, decision number 15-093) as well as Informed Consent from the families of tissue donors according to the declaration of Helsinki. Was previously obtained.

Human limbal epithelial (HLE) cells were maintained in culture medium consisting of DMEM F12 (1:1) (Life Technologies, Darmstadt, Germany) with added 10% Fetal Bovine Serum, 1% penicillin/streptomycin/amphotericin (Life Technologies, Darmstadt, Germany), 0.1 nM cholera toxin B (Sigma, Munich, Germany), 0.05 mM hydrocortisone (Sigma, Munich, Germany), 5 μg/ml human recombinant insulin (Sigma, Munich, Germany), and 10 ng/ml epidermal growth factor (Life Technologies, Darmstadt, Germany). Culture medium was exchanged three times a week. For the isolation, the tissue was immersed in a 1.2 U/ml dispase II solution (Sigma, Munich, Germany) overnight at 4 °C or for 2 hours at 37 °C. Subsequently, the tissue was placed into a 10 cm petri dish. The epithelial cells were gently removed by scraping using a feathered scalpel and aiming at the limbus to achieve an enriched LESC/progenitor population. The cells were co-cultured in a T-25 tissue culture flask (Nunc, Schwerte, Germany) with growth arrested 3T3 fibroblasts at a cell density of 2.4 × 10^4^ cells/cm^2^ which acted as a feeder layer. The cultures were maintained at 37 °C and 5% CO_2_ in air.

### Isolation and culture of human limbal fibroblasts

Limbal fibroblast cultures were obtained by human corneo-scleral rims and buttons after obtaining Ethics Approval (State of Cologne Ethics Approval Committee, decision number 15-093) and Informed Consent from the families of tissue donors (according to the declaration of Helsinki).

Following the harvesting of the epithelial cells for culture, as described above, the scleral and corneal tissues were trimmed to leave approximately 1 mm on either side of the limbus. Then, the tissue was further cut into smaller pieces. These fragments were allowed to stick on to 10 cm petri dishes. The explants were maintained in DMEM (life technologies, Darmstadt, Germany) plus 10% FBS and 1% penicillin–streptomycin (life technologies, Darmstadt, Germany) until fibroblasts grew out. The cells were subcultured at a ratio of 1:2 and the medium was exchanged every 48 h.

### Experimental Setup – ACUVUE OASYS^®^ contact lenses (senofilcon A)

**Figure 1:**
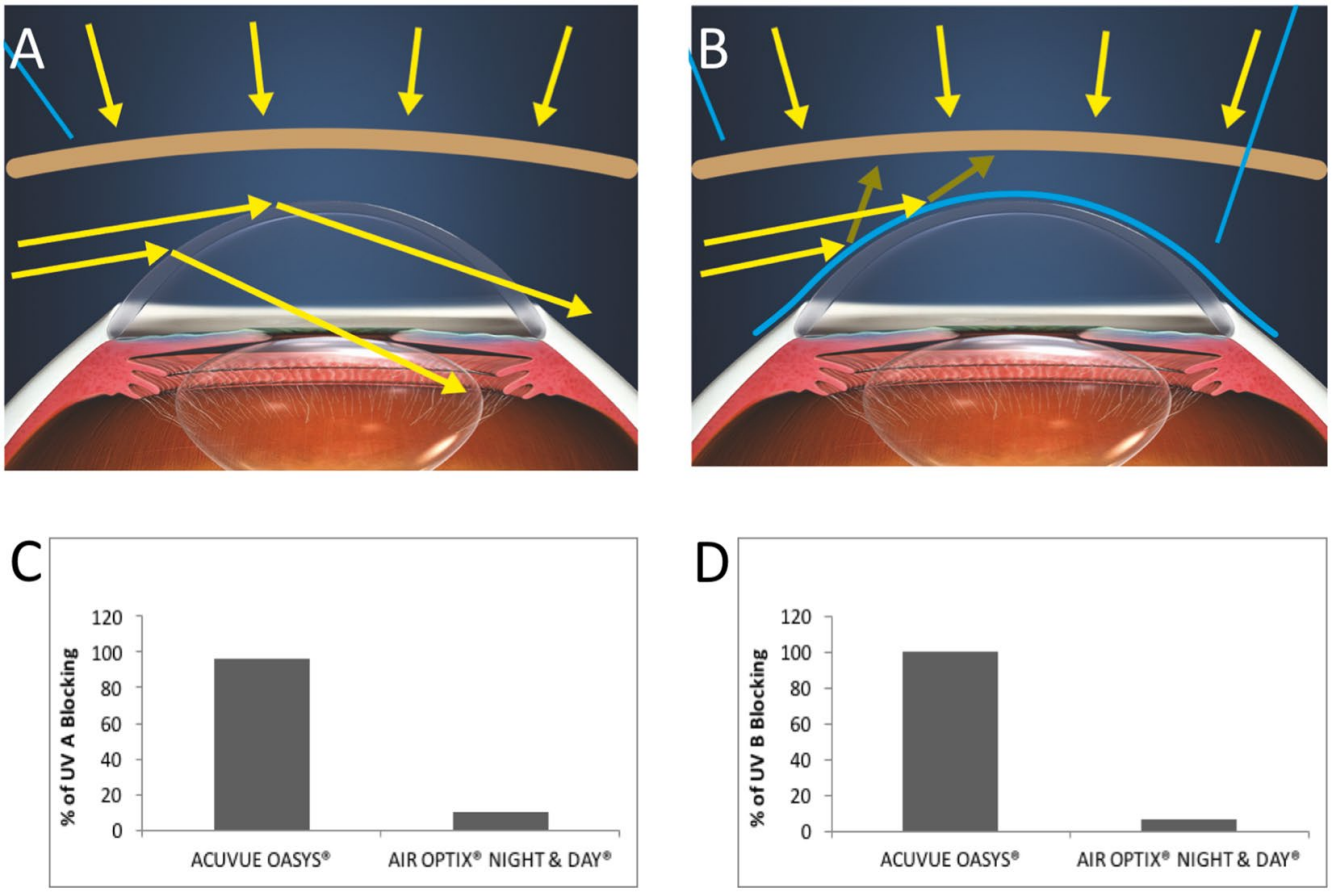
UVB blocking contact lenses effectively block UVA and UVB light. (**A**) Protective UV-absorbing eyewear, such as sunglasses, alone are not able to protect the ocular surface from UV rays coming from the side direction (depicted in yellow arrows) (**B**). UV blocking contact lenses offer total protection of the cornea and the limbus and when combined with UV protective eyeware the entire ocular surface is protected. The UV blocking contact lenses tested, ACUVUE OASYS (senofilcon A) offer 10-fold more protection for UVA and 17-fold for UVB, reaching 96% and 100% blocking of UVA (**C**) and UVB (**D**) respectively compared to contact lenses without a UV blocker, namely Air Optix Night & Day (lotrafilcon A). Graphics with courtesy from Johnson & Johnson Vision.

While protective UV-absorbing eyewear, such as sunglasses, alone are not able to protect the ocular surface from UV rays coming from the side direction (Fig. 1A), in combination with UV blocking contact lenses, they are able to offer total protection of the cornea and surrounding area (Fig. 1B). The UV blocking contact lenses tested were ACUVUE OASYS (senofilcon A, Johnson & Johnson Vision, Florida, USA) with a Class 1 UV blocker. When compared to contact lenses without UV blocker such as Air Optix Night & Day (lotrafilcon A, Alcon, Texas, USA), they offer 10-fold more protection for UVA and 17-fold for UVB reaching 96% and 100% blocking of UVA (Fig. 1C) and UVB (Fig. 1D) respectively.

Limbal epithelial cells or limbal fibroblasts were cultured in 48-well plates at a density of 10^5^ cells per well in a minimum of 4 replicates in columns. One row was left empty between each treatment group. The diameter of the lenses is 3 mm larger than the diameter of the plate wells to ensure complete coverage of the cultures.

After 24 h, the culture medium was exchanged with PBS and the plate was coated with AbsorbMax film (an adhesive material which blocks UV light from Sigma, Munich, Germany) on all available surface with the exception of the wells which would be covered with the two contact lens types and the wells with the uncovered cells control. Special care was given to applying the film in spaces between the wells to reduce stray light and well-to-well crosstalk. The cell cultures were either covered with AbsorbMax (BT), covered by UV-blocking contact lenses (group labelled UVBCL, ACUVUE OASYS or covered by contact lenses without UV protection (group labelled CL, Air Optix Night & Day) or unprotected (group labelled NO COVER). Non-irradiated cultures in a separate plate were used as an additional control (group labelled NO UV). The alamar blue assay was carried out 24 h post-irradiation with 50mJ/cm^2^ UVB for the Alamar Blue assay only and 20mJ/cm^2^ for all other experiments using a VilberLourmat (Eberhardzell, Germany), Bio-Sun UV irradiator using a 2 W 312 nm. An automatic sterilisation program using a germicidal lamp at 254 nm which was used prior to placing the plate in the irradiator to ensure that no contamination would occur. The software was set at UVB and the required dose was predefined depending on the experiment.

### Cell Metabolic Activity

Cell metabolic activity was assessed by using the Alamar blue assay (Thermo Scientific, Schwerte, Germany). Specifically, the cultures were incubated for 1 h in 300 μl/well alamar blue reagent in a 1/10 ratio in PBS. A minimum of 5 replicated were used. Cell-free wells containing alamar blue reagent were used as blanks. The plates were analysed in an Epoch plate reader (BioTek, Bad Friedrichshall, Germany) in absorbance mode at 570 nm and 600 nm. The percentage of reduction of the alamar blue reagent was calculated as described in the manufacturer’s instructions. These experiments were repeated with cells from a minimum of 3 different donors.

### Scratch wound assay

Scratch wound assays were performed, as previously described^20,21^. Briefly, the cells were plated in completely confluent layers in 48 well plates and treated with UVB using the set-up described in the section above this time with UVB 20mJ/cm2. Subsequently, a scratch was inflicted by using a 10 μl pipette tip (*n* = 8). The wounds were photographed at 0, 4 and 8 h. The wound areas at each time-point were calculated by using Image J software. The data were presented as a percentage of healed wound area compared to the original wound area at time point 0 h. The experiments were replicated at least 3 times with cells from different donors.

### UV-irradiation of cells and collection of conditioned medium for ELISA

The cells were plated to 90% confluence in 48 well plates. To avoid the use of feeder cells, HLE cells were first expanded as described in the sections above and then separated from the 3T3 cells by using differential trypsinisation. Subsequently, they were plated in a serum-free and feeder-free corneal epithelial culture media CNT-57 (CellnTech, Bern, Switzerland). The HLF cells were placed in their usual culture media (DMEM supplemented with 10% FBS and 1% pen-strep). Prior to irradiation, the culture media was replaced with PBS. The cultures were irradiated at 20mJ/cm^2^ by using a Vilber Lourmat (Eberhardzell, Germany) Bio-Sun UV irradiator set at 265 nm. The PBS was replaced with MV2 basal endothelial medium (Promocell Heidelberg, Germany) supplemented with 2% FBS (basal medium, BM). The produced conditioned media were collected after 24 h, centrifuged at 1500 G to clear from dead cells and debris, aliquoted and stored at −80 °C for a maximum of 2 months before use for analysis.

### Immunocytochemistry of cells

The cells were cultured in eight-well permanox chambered slides (labtek, Nunc, Schwerte, Germany), rinsed three times with PBS and treated for 10 minutes at room temperature in 4% (wt/vol) paraformaldehyde to fix. The samples were blocked for 1 h in PBS with added 5% goat serum (Sigma, Munich, Germany) and 0.5% Triton X (Sigma, Munich, Germany) followed by the primary antibody (mouse anti-ABCB5 monoclonal antibody clone 3C2-1D12^22,23^ rabbit polyclonal integrin beta 1 antibody from Abcam (Cambridge, UK), mouse monoclonal antibody for cytokeratin (K)3 from Millipore (Darmstadt, Germany) and rabbit polyclonal antibody for P63α from New England Biolabs (Frankfurt am Main, Germany) overnight at 4 °C. Subsequently, the specimens were incubated with their respective secondary antibody (goat anti-rabbit alexa 488, goat anti-mouse alexa 647, both from Life Technologies, Darmstadt, Germany), rinsed and stained with DAPI (Sigma, Munich, Germany). All incubations, except the primary antibody incubation, were performed at room temperature, and each step was intermittent with 3 × 5 minute washes with PBS containing 0.1% tween-20 (Sigma, Munich, Germany). Negative controls were treated in the same way except skipping the primary antibody step. A minimum of 3 random fields of each stained sample were photographed. The percentages of marker-positive cells were measured by using the plugin ‘cell count’ of Image J (DAPI was used to count the total number of cells). The experiments were repeated with cells from at least 3 different donors.

### Colony forming efficiency assay

For the colony forming efficiency (CFE) assay^24,25^, 3T3 fibroblasts were used as a feeder layer. The cells were growth arrested by using mitomycin C (Sigma, Munich, Germany) as already described and seeded at a cell density of 4.8 × 10^5^ cells per well of a six well plate. HLE were plated at a clonal cell density of 1000 cells per well of the six well plate. After 12 days, the cultures were fixed with cold methanol for 20 min at −20 °C. Subsequently, the cells were treated with a solution of 1% rhodamine B (Sigma, Munich, Germany) and 1% Toluidine Blue (Sigma, Munich, Germany) for 30 min at 37 °C. The plates were photographed and Image J software was used to count the number of colonies that measured higher than 2 mm diameter. The percentage of colony forming efficiency was calculated by using the equation:

$${\rm{CFE}}( \% )=\frac{{\rm{Number}}\,{\rm{of}}\,{\rm{colonies}} > 2{\rm{mm}}}{{\rm{Number}}\,{\rm{of}}\,{\rm{cells}}\,{\rm{seeded}}}\times 100$$

The experiments were performed with cells from a minimum of three donors (technical replicates n = 6).

### ELISA

Quantikine ELISA kits for TNFα, MCP1, IFNγ, VEGFA and VEGFC from R&D biosciences (Wiesbaden-Nordenstadt, Germany) were used for protein quantification following the manufacturer’s instructions. Each sample was analyzed in duplicate. The proteins were quantified in conditioned medium from a minimum of three donors.

### Statistical analysis

Statistical analysis was performed by using Prism 6.0 software (GraphPad). One-way Analysis of Variance (ANOVA) with Tuckey’s multiple comparisons test was used. Statistically significant differences were considered between sets of data producing *p* < 0.05. The experiments were carried out using a minimum of 3 technical triplicates and repeated at least three times by using cells from at least three different donors. All error bars represent values of standard deviation (SD).

### Data availability Statement

The datasets generated during and/or analysed during the current study are available from the corresponding author on reasonable request.

## Results

### UV blocking contact lenses prevent UVB-induced cyclobutane pyrimidine dimer formation in limbal epithelial cells and fibroblasts

**Figure 2:**
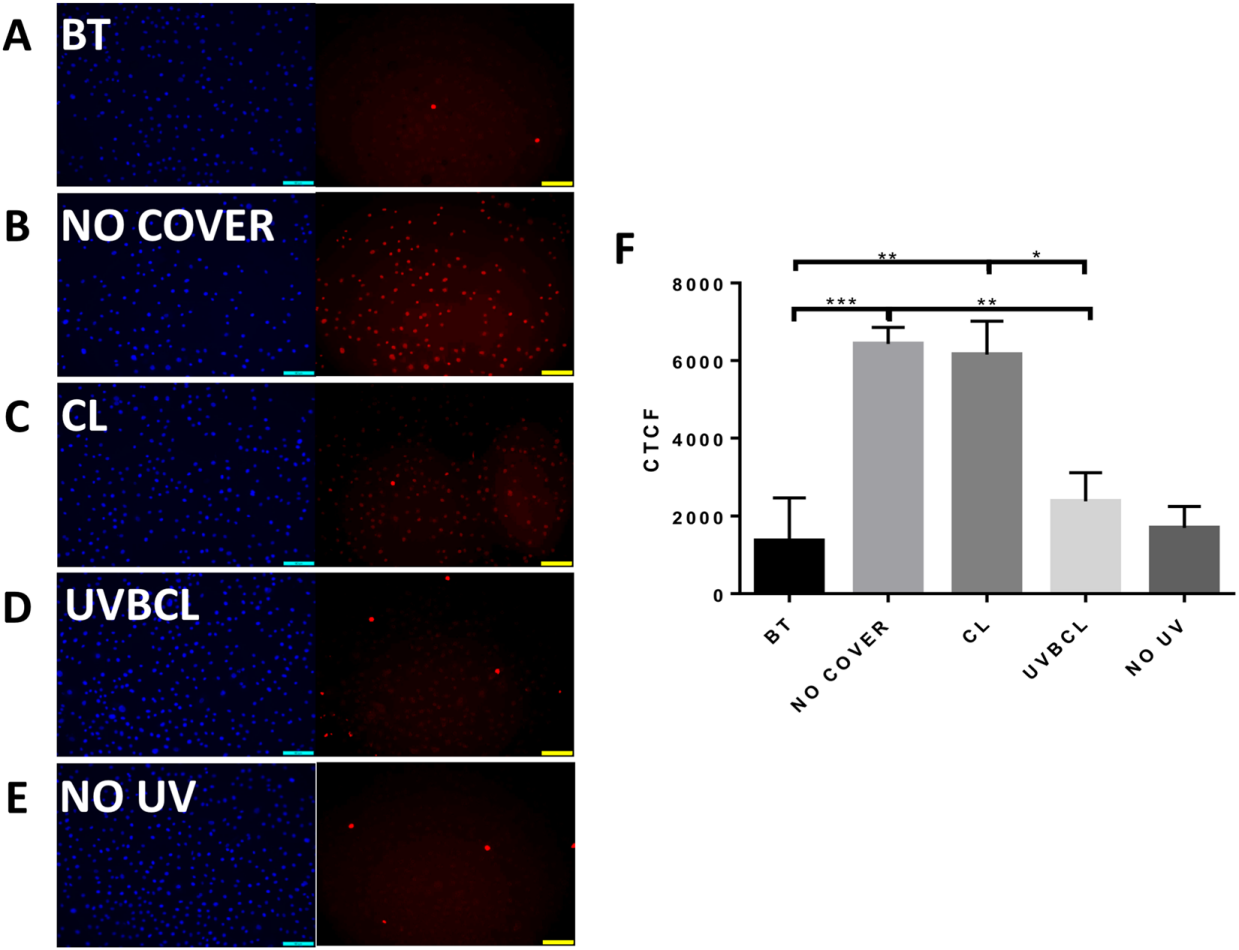
Senofilcon A UVB blocking contact lenses prevent UVB-induced formation of cyclobutane pyrimidine dimers (CPD, marker of DNA damage) in limbal epithelial cells. Representative immunofluorescence photos of CPD in limbal epithelial cells following UVB irradiation (20 mJ/cm^2^): BT (AbsorbMax black tape) (**A**) NO COVER (**B**), CL (**C**), UVBCL (**D**) and NO UV (**E**). (**F**) Quantified corrected total cell fluorescence (CTCF, n = 6, *p < 0.05, **p < 0.01, ***p < 0.001).

**Figure 3:**
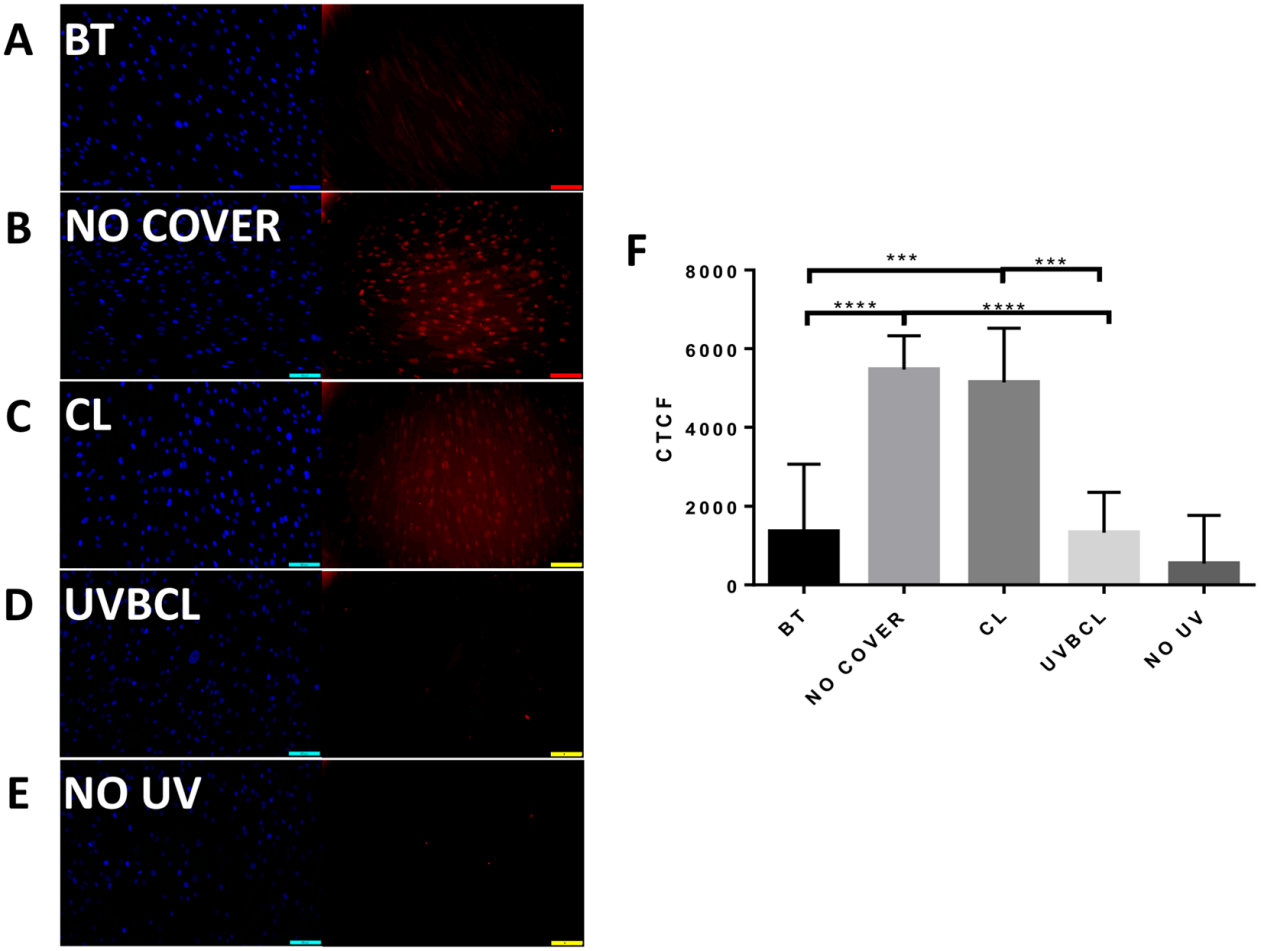
Senofilcon A UVB blocking contact lenses protect against UVB-induced cyclobutane pyrimidine dimer (CPD) formation in limbal fibroblasts. Representative immunofluorescence photos of CPD in limbal fibroblasts following UVB irradiation (20 mJ/cm^2^): BT (AbsorbMax black tape) (**A**) NO COVER (**B**), CL (**C**), UVBCL (**D**) and NO UV (**E**). (**F**) Quantified corrected total cell fluorescence (CTCF, n = 6, ***p < 0.001, ****p < 0.0001).

In order to investigate the protective effect of the UVBCL protective contact lenses against direct DNA damage, the formation of cyclobutane pyrimidine dimers (CPD) was assessed immediately following UVB exposure of HLE and HLF cells. Cells protected with a UV blocking adhesive film (BT) and cells not exposed to UVB irradiation were used as negative controls, while cells directly exposed to UVB were used as positive controls. CPD formation was visualized using a specific antibody and the corrected total cell fluorescence (CTCF) was quantified with Image J. The immunofluorescence data of HLE cells showed that the UV absorbing film (Fig. 2 BT, A) minimized CPD formation to a level comparable to the cells that did not receive any irradiation (Fig. 2, NO UV, E). The CL contact lenses reduced the CPD incidence and intensity (Fig. 2, CL, C); however, the UVBCL contact lenses (Fig. 2, UVBCL. D) diminished the CPD signal to levels similar to No UV and BT. The CTCF quantification also showed that CPD levels in CL where significantly higher than UVBCL (F, p < 0.05) while UVBCL was not significantly different than BT and NO UV. The NO COVER group was significantly higher than all other groups. The same trends were observed in differently treated groups of HLF cells (Fig. 3F and representative immunofluorescence photos of BT (A) NO COVER (B), CL (C), UVBCL (D) and NO UV (E)).

### UV blocking contact lenses maintain metabolic activity and rate of scratch wound closure of limbal epithelial cells and fibroblasts

To investigate the effect of UVB irradiation in HLE and HLF cells we used a higher dose of UVB irradiation (50 mJ/cm^2^) as we have shown before that the dose 20 mJ/cm^2^ is not causing changes in cell viability and proliferation^20^ (and Supplementary Figure 1).

**Figure 4:**
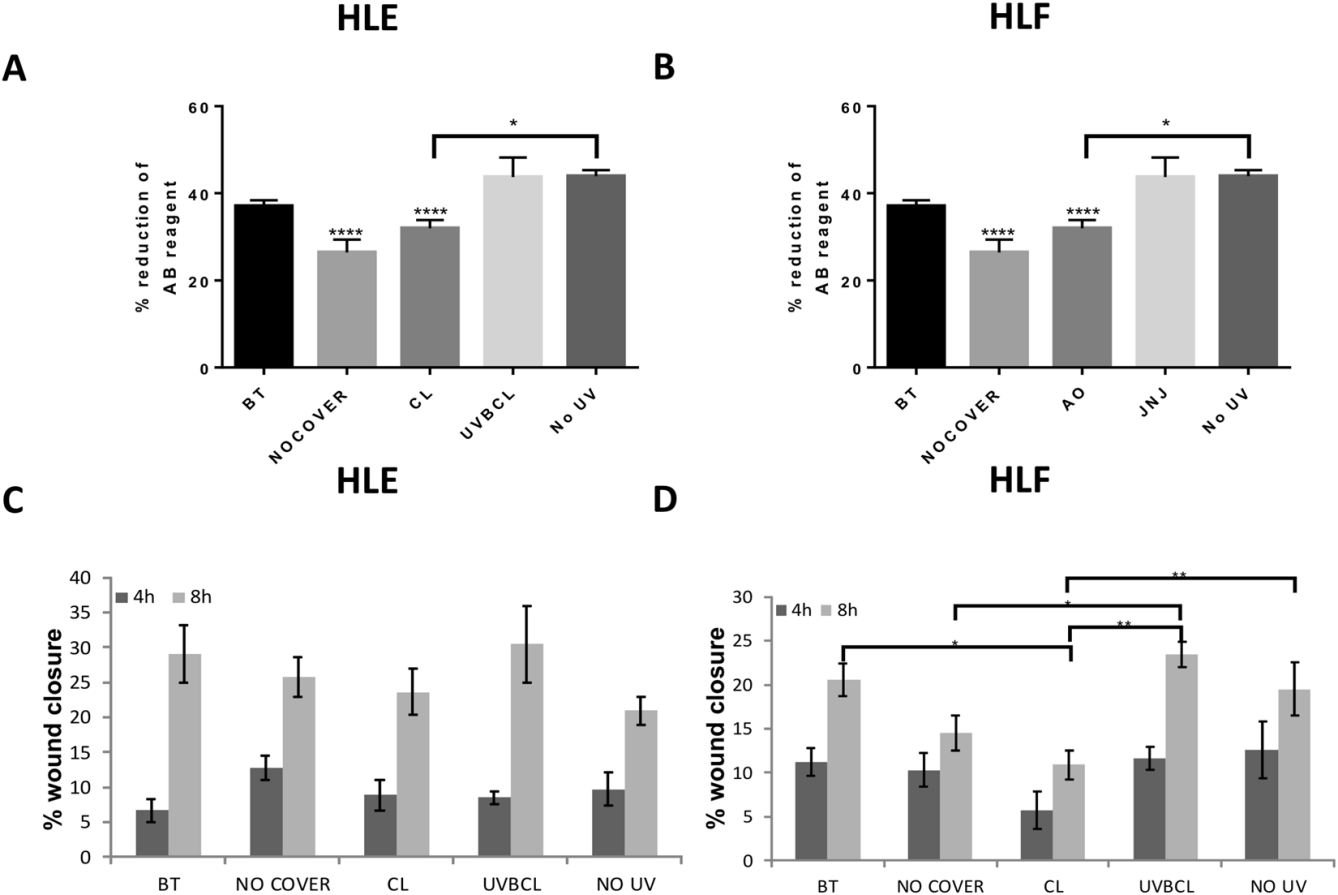
Senofilcon A UVB blocking contact lenses protect against changes in the metabolic activity and rate of scratch wound closure of limbal epithelial cells and fibroblasts. (**A**,**B**) Alamar Blue data showed that the metabolic activity of HLE (**A**) and HLF (**B**) cells which were protected by the UVBCL contact lens was maintained in similar levels to the BT and NO UV controls (20 mJ/cm^2^). (**C**,**D**) HLE cells exhibited non-significant changes in wound healing activity at neither 4 h nor 8 h time-point following UVB irradiation (**C**). HLF cells protected by CL contact lenses featured slower wound closure compared to cells covered by UVBCL lenses, BT and cells that were not UV treated (NO UV) (**D**). n = 6, *p < 0.05, **p < 0.01, ***p < 0.001, ****p < 0.0001.

Alamar Blue data showed that the metabolic activity of both HLE (Fig. 4A) and HLF (Fig. 4B) completely unprotected cells was significantly reduced following UVB irradiation (p < 0.0001 from all other groups in both cell types). When covered with the CL contact lens both HLE and HLF cells exhibited higher viability than the uncovered cells, however, the difference was not statistically significant. In contrast, the UVBCL UV-blocking contact lenses maintained similar levels of metabolic activity with the BT and No UV controls. The difference between CL and UVBCL lenses was significant in both cell types (p < 0.0001).

A scratch wound assay was carried out to illustrate differences in migration between different groups. The test was carried out following irradiation treatment with 20 mJ/cm^2^ UVB to avoid discrepancies owing to effects on proliferation and viability. Regarding the response of HLE cells, we found that there were no significant effects at either a 4 h or 8 h time-point following treatment (Fig. 4C). HLF cells on the other hand exhibited statistically significant differences between groups at the 8 h time-point (Fig. 4D). Specifically, the CL contact lenses HLF group displayed slower scratch closure compared to UVBCL lenses (p < 0.01) as well as compared to the BT (0.05) and NO UV (p < 0.01) cell groups. The UVBCL group was comparable to the NO UV and BT groups (Fig. 4D).

### UVB-induced alterations in the limbal epithelial cell progenitor-like phenotype are prevented with UV blocking and protection by UVBCL contact lenses

**Figure 5:**
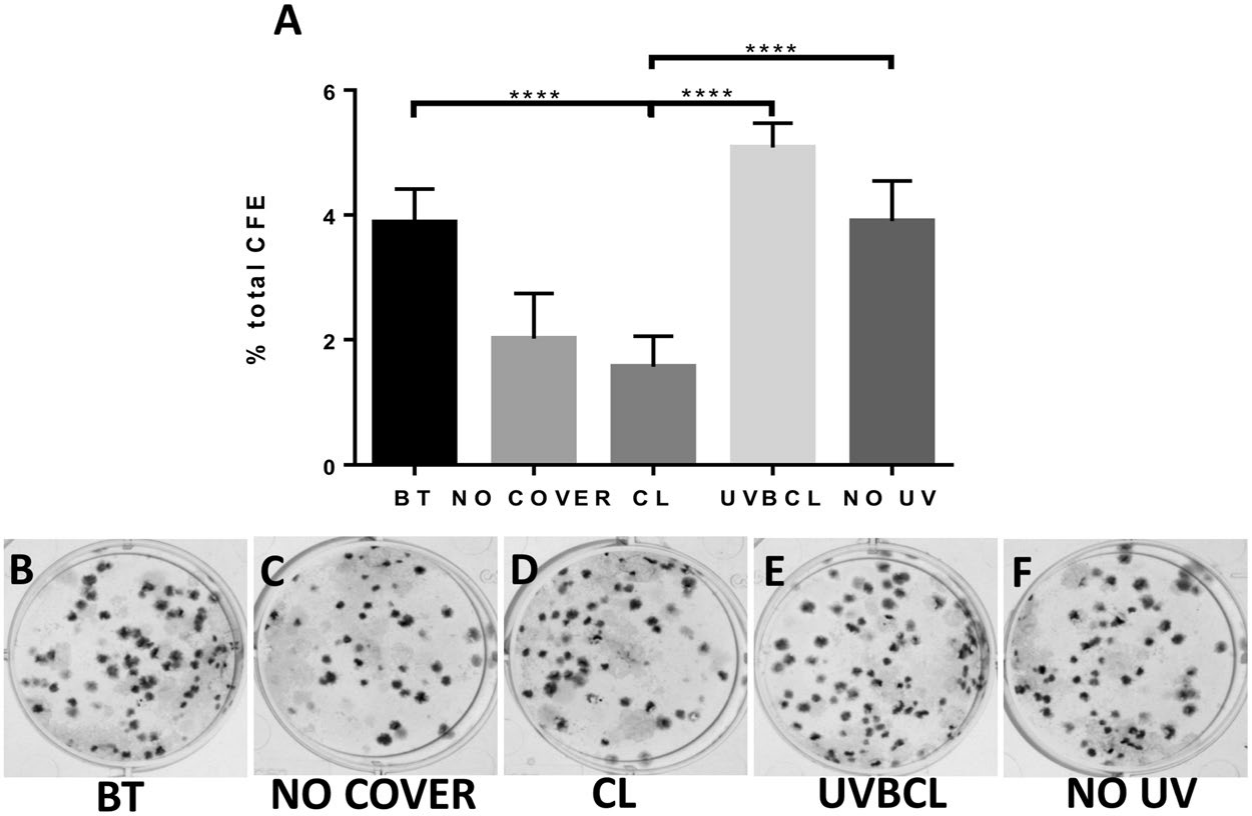
UVB-induced reduction of limbal epithelial cell colony forming efficiency is prevented with protection by senofilcon A UVB blocking contact lenses. (**A**) Colony forming efficiency of irradiated limbal epithelial cells, either unprotected or covered with CL lenses, was significantly reduced compared to their non-irradiated counterparts indicating loss of proliferative potential as a result of ultraviolet B treatment. This is reversed by using UVBCL contact lenses to protect the HLE cells from UVB irradiation. Photos B-F depict representative photos of cultures, n = 6, ****p < 0.001.

**Figure 6:**
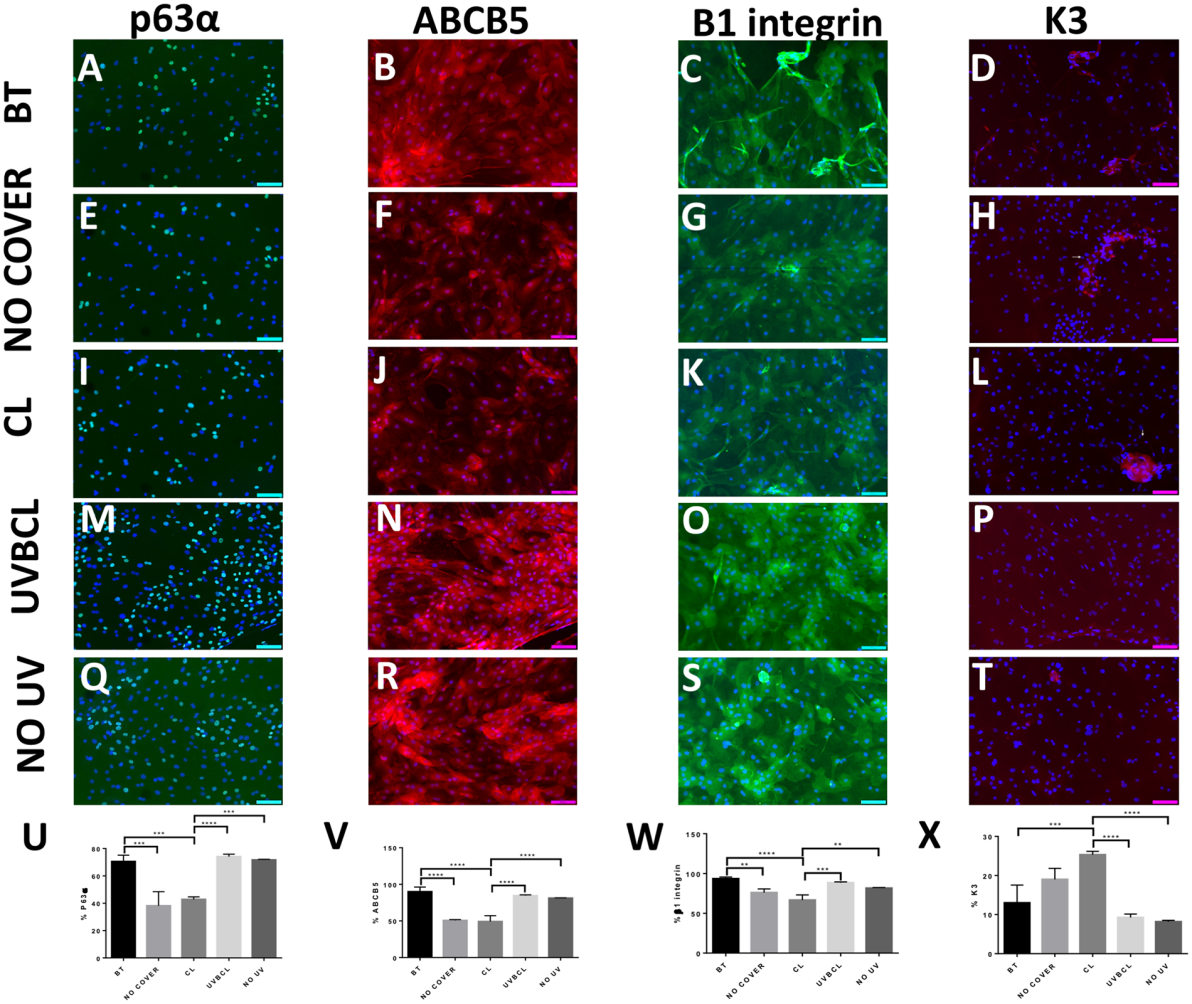
UVB-induced changes of limbal epithelial cell phenotype is prevented with protection by senofilcon A UVB blocking contact lenses. Immunocytochemistry of limbal epithelial cell marker expression including p63α (**Α**,**E**,**I**,**M**,**Q**, Alexa488), ABCB5 (**B**,**F**,**J**,**N**,**R**, Alexa 555), β1 integrin (**C**,**G**,**K**,**O**,**S**, Alexa488) and keratin 3 (**D**,**H**,**L**,**P**,**T**). Limbal epithelial cells in the NO COVER and CL groups partially lost the expression of the markers integrin p63α (**E**,**I**), ABCB5, (**F**,**J**) β1integrin (**G**,**K**) while areas expressing the differentiation marker K3 increased (**H**,**L**, white arrows accentuated K3-positive regions). The immunocytochemistry data are quantified and summarized in graph (**U**,**V**,**W**,**X**), n ≥ 3, **p < 0.01, ***p < 0.001, ****p < 0.0001. Scale bars correspond to 100 μm.

Together with the UVB blocking and protective effect of UVBCL contact lenses on HLE proliferation, UVBCL contact lenses were also able to reverse changes in stem cell phenotype. Specifically, the colony forming efficiency of the CL cells significantly declined compared to the NO UV and UVBCL cell groups (approximately 2-fold decrease, p < 0.0001, Fig. 5A). At the same time, the UVBCL CFE levels were similar to the NO UV and BT controls (Fig. 5A, panels B-F represent the CFE cultures from all treatment groups). Immunocytochemistry data of CL and NO COVER HLE cultures featured areas with a differentiated morphology consisting of enlarged cells which did not express the stem cell markers P63α, ABCB5 and integrin β1 (Fig. 6 panels E/I, F/J and G/K respectively). In addition, these cultures exhibited an increase in K3-positive cells or cell clusters (Fig. 6 L/P). The quantified immunocytochemistry data (depicted in Fig. 6 U-X) illustrated a significant reduction in P63α-, ABCB5- and β1 integrin-positive cells in the CL group compared to the UVBCL group (Fig. 6U, p < 0.0001, Fig. 6V, p < 0.001 and Fig. 6W, p < 0.001) as well as the BT (Fig. 6U, p < 0001, 6 V, p < 0001, 6 W, p < 0.001) and NO UV (Fig. 6U, p < 0.001, 6 V, p < 0.0001 and Fig. 6W, p < 0.01) controls. In contrast, the number of K3 positive cells in CL cultures was significantly higher compared to the UVBCL group (Fig. 6X, p < 0.0001) as well as the BT and NO UV controls (Fig. 6X, p < 0.001 and p < 0.0001).

### UV-blocking contact lenses prevent UVB-induced changes in secreted pro-inflammatory and pro-angiogenic cytokines

**Figure 7:**
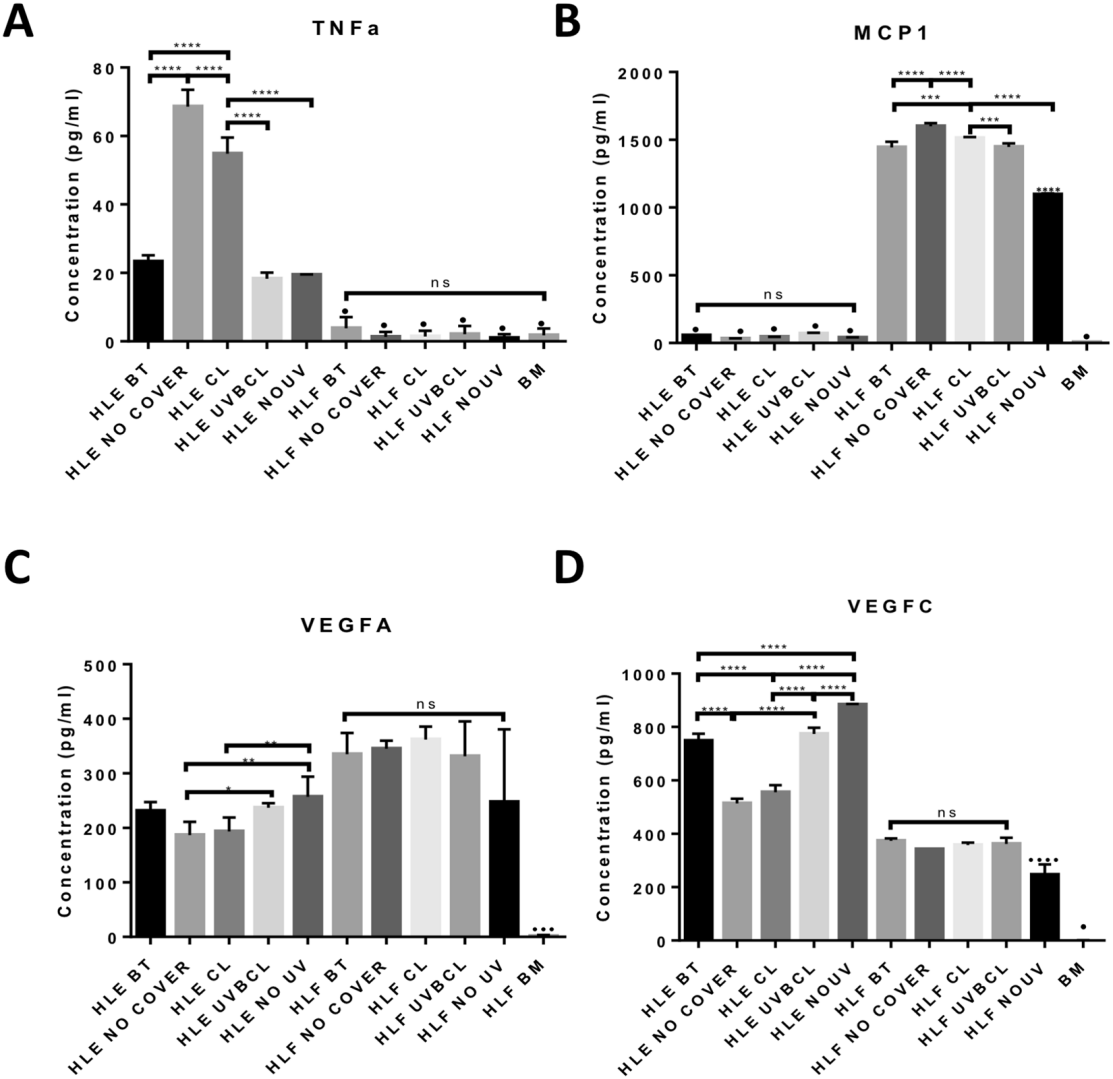
Senofilcon A UVB blocking contact lenses prevent modifications in the expression of key inflammation and angiogenesis-related proteins produced by limbal epithelial cells and limbal fibroblasts. ELISA analysis of conditioned media from limbal epithelial cells and limbal fibroblasts for TNFα (**A**), MCP-1 (**B**), VEGFA (**C**) and VEGFC (**D**). UVB irradiation induced an upregulation of TNFα and MCP-1 (in limbal epithelial cells and limbal fibroblasts respectively) while this was prevented by the use of UVBCL lenses to protect the cultures (**A**,**B**). UVB irradiation of limbal epithelial cells induced a reduction in VEGFA and VEGFC (**C**,**D**) while covering of the cultures with UVBCL lenses maintained the protein levels similar to the ones of the controls. (n = 3, *p < 0.05, **p < 0.01 and ***p < 0.001 and ****p < 0.0001). Four asterisks (****) situated above bars without brackets correspond to significance in comparison to all other groups. (● signifies that the protein levels were under the detectable levels of the assay).

ELISA analysis of supernatants produced by HLE and HLF cells of different treatment groups was carried out so as to assess their potential paracrine pro-inflammatory and pro-angiogenic effects. Notably, none of the proteins examined was detected in the BM (Fig. 7A–D).

Analysis for TNFα showed a (3-fold) significant increase of its levels in the NO COVER and CL groups compared to the NO UV and the BT groups (Fig. 7A, p < 0.0001). Even though there was a small but significant reduction of TNFα production by the CL group compared to uncovered cells (from 70 to 55 pg/ml, p < 0.0001), it was still significantly 3-fold higher than in the UVBCL group (p < 0.0001). The protein level for the UVBCL group was similar to the NO UV and BT controls. Regarding HLF cells, TNFα concentrations were under 4 pg/ml on average and significantly lower compared to all HLE groups (p < 0.0001). There was no significant difference in the TNFα expression between all HLE groups.

The assessment of MCP1 levels in the supernatants from the various treatment groups showed that the protein was produced in significantly higher (by over 20-fold) amounts by HLF cells compared to HLE cells (Fig. 7B, p < 0.0001), while there was no significant difference between the different HLE cell groups. A significant increase of the protein concentrations was noted in the NO COVER and CL groups compared to the NO UV and the BT groups (Fig. 7B, p < 0.0001). While there was a small but significant reduction of the produced cytokine by the CL group in relation to the uncovered cells (p < 0.0001), levels were still significantly higher than in the UVBCL group (p < 0.001). Although the protein level for the UVBCL group was similar to the BT controls, it was still significantly higher compared to the NO UV group.

VEGFA levels produced by HLE cells where significantly lower in the CL group compared to the NO UV control group (Fig. 7C, p < 0.01), while the NO COVER HLEs produced lower levels compared to UVBCL and NO UV HLEs (7 C, p < 0.05 and p < 0.01 respectively). No significant differences were observed between the HLF treatment groups.

VEGFC was produced in higher (approximately 2-fold) amounts by HLE cells as opposed to HLF cells (Fig. 7D, p < 0.0001) while there was no significant difference between the different HLE cell groups, with the exception of the No UV group that exhibited a small but significant decrease compared to the other HLF treatments (p < 0.0001). A significant decrease of the protein concentrations was noted in the NO COVER and CL groups compared to the NO UV and the BT groups (Fig. 7D, p < 0.0001). There was a small but not significant difference between the CL group and the uncovered cells, while the level was still significantly lower than in the UVBCL group (p < 0.0001). Although the VEGFC output for the UVBCL group was similar to BT controls, it was still significantly lower compared to the NO UV group (p < 0.0001).

## Discussion

The experiments presented here allow for the following conclusions to be drawn:

(i) UVB irradiation leads to loss of function of limbal epithelial cells, fibroblasts as well as loss of stemness of epithelial progenitor cells and ability to form holoclones. In addition, UVB irradiation leads to a pro-inflammatory milieu *in vitro*.
(ii) These damaging and pro-inflammatory effects can, to a great extent, be prevented by a UVB blocking contact lens as opposed to contact lenses without a UV-blocker.
(iii) This suggests the use of UV blocking contact lenses to be useful, especially in the context of e.g. pterygium recurrence and after limbal stem cell transplantation.

Our group and others have previously reported that UVB irradiation has a profound effect on phenotype and functionality of cellular components of the limbal stem cell niche^20,26–30^. Specifically, we demonstrated that short term UVB irradiation reduced putative stem cell marker expression and CFE of irradiated limbal epithelial cells. At the same time, UVB triggered the production of pro-inflammatory and macrophage attracting cytokines by both limbal epithelial cells and fibroblasts thus upsetting the balance which maintains corneal avascularity and lack of inflammation^20^. For that reason, protective strategies against UVB should be beneficial towards maintaining limbal stem cell niche homeostasis. The use of protective equipment to prevent eye exposure to UV irradiation is widely recommended as long term exposure is linked to conditions such as acute keratitis^31^, cataract^32^ and pterygium^33^. With the exception of a few reports showing that UV blocking contact lenses have been proven preventative against acute photo-keratitis caused by UVR overdoses in animal models^2,3,34–37^, the precise effect of these lenses remains unknown.

The data presented in this study demonstrated a substantial protective effect of UV blocking contact lenses (UVBCL) against UV damage in both limbal epithelial cell and fibroblasts compared to conventional contact lenses and controls. The UV-induced formation of 6-4 photoproducts and cyclobutane pyrimidine dimers is normally restored by a nucleotide excision repair (NER) mechanism which removes bulky DNA lesions to restore the canonical nucleotide sequence^38^. CPD are the most mutagenic UV-induced DNA adducts^39^ and are responsible for C → T and CC → TT transition mutations at dipyrimidinic sites, the signature mutations induced by UV light^40^. Previous reports indicate that UVB induced CPDs occur in all corneal layers^41^ although they are repaired faster in comparison to epidermal keratinocytes thus suggesting that DNA repair mechanisms might be more active in the corneal epithelial cells compared to the skin epithelium^42^. Our data confirm that the senofilcon A UVBCL UV blocking contact lens was able to prevent the occurrence of UVB damage in the form of CPDs in both limbal epithelial cells and fibroblasts.

In addition to DNA damage, previous *in vivo* as well as *in vitro* studies have demonstrated that UVB exposure of the cornea directly affects cell viability and wound healing^43–45^. UVB-mediated apoptosis can take place via K^+^ mediated caspase 3 and 8 activation (extrinsic pathway) or mitochondrial death induced activation of caspase-9 (intrinsic pathway)^46,47^. Additionally, corneal epithelial cell apoptosis is regulated by p53 which becomes activated as a result of CPDs and other DNA damage^42^. We have illustrated that the protective effect of the senofilcon A UV blocking contact lenses restored viability in epithelial and fibroblasts cultures. The ability of UVBCL lenses to prevent the formation of CPDs in limbal epithelial cells and fibroblasts may therefore be contributing towards maintenance of cell viability.

The scratch wound healing rate of limbal fibroblasts also decreased following UVB-treatment, while the use of UVBCL lenses restored it to the levels of the controls. It should be noted that the scratch wound closure assay was carried out following UVB exposure of 20mJ/cm^2^, which we show here not affect cell viability. Therefore, the observed differences in wound healing rates were owing to an effect of irradiation on cell function and not a reduction of cell numbers. Although the assay did not show significant differences between groups in epithelial cells, the HLF cells exhibited a reduction of their scratch healing rate following irradiation, confirming previous studies, that reported similar effects in dermal fibroblast cells *in vitro* and *in vivo*^48,49^. UV-protection of HLF cultures with the UVBCL contact lens maintained wound healing rates similar to the controls.

Our group has recently demonstrated the effect of UVB on HLE and HLF cell functionality and phenotype^20^. Here we confirmed that HLEs that were UVB-treated or were protected by the UVBCL lenses displayed the typical phenotype of small cobblestone-like, tightly packed cells which expressed putative LESC markers, including P63a^50^, ABCB5^23^ and β1 integrin^51^. A significant loss of these markers is observed in CL and NO COVER cell groups, coinciding with an increase in K3-positive cell clusters and a significant drop in CFE. These changes indicate loss of stem cell phenotype following UVB irradiation.

After investigating the direct impact of UVB on limbal epithelial cells and fibroblasts, conditioned media from all treatment groups was collected to assess the secretion of pro-inflammatory and pro(lymph)angiogenic cytokines previously found to change in HLE and HLF cells upon UVB treatment^20^. ELISA data showed that VEGFA and VEGFC were significantly increased in No COVER and CL cells, while the UVBCL levels were similar to the BT control. Changes in the produced inflammatory and macrophage-attracting cytokines were also assessed. UVB irradiation induced upregulation of the pro-inflammatory cytokines TNFα (HLE) and MCP1 (HLF) of NO COVER and CL cells, while cytokine levels in UVBCL and control cells were similar. TNFα is a key inflammatory factor^52^ known to regulate leukocyte recruitment, vasodilatation and edema, all of which are likned to cornea neovascularization^53^. MCP1 is both a pro-angiogenic chemokine^54^ and a major monocyte attractor^55^. It is therefore here demonstrated that UVBCL lenses may prevent an increase of these cytokines in HLE and HLF cells, which may contribute to the inflammatory reactions in the cornea following UVB irradiation.

Overall, our data confirmed that the use of senofilcon A, Class 1, UVB blocking contact lenses may prevent short term UVB exposure-induced DNA damage, limbal epithelial cell differentiation and secretion of pro-inflammatory cytokines in cultured limbal epithelial cells and fibroblasts. The use of these lenses in a clinical setting may prove beneficial against pterygium recurrence and as a prophylactic measure in patients receiving cultured limbal stem cell graft transplantation.

## Electronic supplementary material

Supplementary figure 1
