# Supplementary material for: UV light-blocking contact lenses protect against short-term UVB-induced limbal stem cell niche damage and inflammation
Source: Sci Rep. 2018 Aug 22;8:12564. doi: 10.1038/s41598-018-30021-8 (PMC6105637; doi:10.1038/s41598-018-30021-8)
Supplement: Supplementary file 1 — Supplementary figure 1 [file 41598_2018_30021_MOESM1_ESM.pdf]

Supplementary Figure

UV light-blocking contact lenses protect against short-term UVB-induced limbal stem cell niche damage and inflammation

Notara M[1], PhD; Behboudifard S[1], Ms; Kluth MA[2, 3], PhD; Maßlo C[2, 3], PhD; Ganss C[2, 3], MD; Frank MH[4, 5, 6], MD; Schumacher B[7, 8], PhD; Cursiefen C, MD[1, 8]

1. Dept. of Ophthalmology, University of Cologne, Germany

2. TICEBA GmbH, Im Neuenheimer Feld 517, Heidelberg, Germany

3. RHEACELL GmbH & Co. KG, Im Neuenheimer Feld 517, Heidelberg, Germany

4. Transplant Research Program, Boston Children's Hospital, Harvard Medical School, Boston, MA, USA

5. Harvard Stem Cell Institute, Harvard University, Cambridge, MA, USA

6. School of Medical Sciences, Edith Cowan University, Joondalup, WA, Australia

7. Institute for Genome Stability in Ageing and Disease, CECAD Research Center, Joseph-Stelzmann-Str. 26, 50931, Cologne, Germany.

8. Center for Molecular Medicine Cologne (CMMK), University of Cologne, Germany

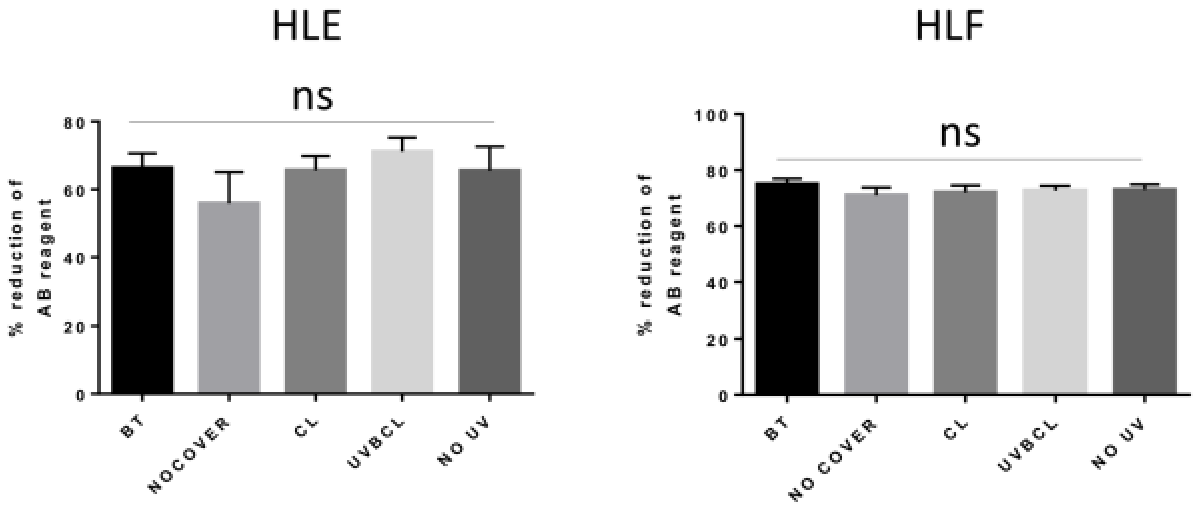

Supplementary Figure 1. The metabolic activity of limbal epithelial cells and fibroblasts remains unchanged following a UVB irradiation dose of 20mJ/cm^2.

Alamar blue assay in limbal epithelial cells (A) and fibroblasts (B), 24h following UVB irradiation demonstrated that cell viability is not affected by a UVB dose of 20mJ/cm2.
